# An effective digital image watermarking scheme incorporating DCT, DFT and SVD transformations

**DOI:** 10.7717/peerj-cs.1427

**Published:** 2023-07-10

**Authors:** Justin Varghese, Omer Bin Hussain, Saudia Subash, Abdul Razak T.

**Affiliations:** 1 Department of Computer Science & Engineering, GITAM School of Technology, GITAM (Deemed to be University), Bangaluru, Karnataka, India; 2 Department of Computer Science, Jamal Mohamed College, Affiliated to Bharathidasan University, Tiruchirappalli, Tamil Nadu, India; 3 Centre for Information Technology & Engineering, Manonmaniam Sundaranar University, Tirunelveli, Tamilnadu, India

**Keywords:** Image watermarking, Singular value decomposition, Image security, Copyright protection, Frequency domain

## Abstract

Image watermarking prevents copyright infringements by attaching visible/invisible watermark images as authentication identities in the owner’s documents. The article made analysis on the advantages of different transformations for choosing better combinations to make the watermark embedding process and observed that watermarking techniques incorporating discrete cosine transform (DCT) provide better resistance towards JPEG based potential attacks, discrete Fourier transform (DFT) has strong energy compaction with geometrical invariance properties to resist geometric attacks while singular value decomposition (SVD) provides stability, proportion invariance and rotation invariance properties and it provides strong resistance against noise based attacks. Considering these advantages of different transformations, the article presents a new non-blind watermarking algorithm by utilizing advantages of DFT, DCT and SVD transforms while attaching secret contents in cover images. The algorithm starts by applying DFT followed by onion peel decomposition (OPD) for decomposing Fourier domain carrier image to four frequency sub images. The scheme then applies DCT on the frequency bands and orders them in zigzag fashion to form four individual frequency arrays. In the final step of embedding process, it embeds four copies of watermark singular value contents in DFT domain with the carrier image singular value contents to produce the watermarked image. The experimental results based on subjective and objective metrics on various test images from standard image databases with different test conditions demarcate the stability of new algorithm in producing high quality watermarked images with fewer distortions even when the watermarked images are extremely distorted by potential attacks.

## Introduction

Extensive and rapid growth of internet based applications and communication technologies have increased vulnerabilities in authenticity, privacy and copyright issues of digital products and addressing these vulnerability issues are prime challenges in cyber space communication applications (Shih, 2017). Transmitted digital data face digital flaws and maltreatment when copied or forged illegally by violating copyrights (Nematollahi, Vorakulpipat & Rosales, 2017). Digital image watermarking deals with attaching visible/invisible watermark images as authentication identities in the owner’s documents (carrier image) for identifying the owner, verifying the authenticity and integrity of the carrier images (Nematollahi, Vorakulpipat & Rosales, 2017). These digital watermarks attached in the owner’s document as metadata are used as the prime forensic information source for tracing copyright violations (Nematollahi, Vorakulpipat & Rosales, 2017; Garg & Rama Kishore, 2020). Digital watermarking techniques are also used for enhancing security in the fields of broadcast monitoring, fingerprinting, indexing, medical imaging *etc*. Intruders/natural processing operations may damage watermarked images leading to removal, detection, overwriting or modification of watermark contents during transmission/unauthorized access. The watermarking methods that attach watermark contents directly to the bits of carrier image can have failures in protecting watermark contents during watermark inversion and other potential attacks necessitating frequency based watermarking approaches that are resistive to potential attacks.

The watermarking techniques are classified in to blind, semi-blind and non-blind approaches according to the additional information needed for reconstructing watermark images with the embedded contents. The full blind watermarking techniques do not need additional information to reconstruct the watermark. The semi-blind schemes require decoding watermark key to recover watermark image. The non-blind approaches require ample amount of original image contents to reconstruct the original watermark from extracted watermark components and it enforces possession of these additional information important in determining the proof of ownership. Hence these methods prevent intruders from recovering the watermark due to non-possession of owner’s private additional information. Additionally, the non-blind approaches as it holds much of the information contents of the watermark as owners proof to recover the watermark, these methods require very less embedding contents in the carrier image when compared to blind schemes that require complete information of the watermarks. This added advantages of non-blind algorithms over blind schemes makes the non-blind schemes produce more appealing watermarked images with better robustness than blind schemes.

The article presents a new non-blind watermarking algorithm by utilizing advantages of discrete Fourier (DFT), discrete cosine (DCT) and singular value decomposition (SVD) transforms while attaching secret contents in cover images. The article is presented in five sections. Section 2 makes the literature survey of the field. Section 3 discusses new watermarking scheme while section 4 makes experimental analysis of the algorithm. Conclusions with future scope are done in section 5.

## Literature survey

Digital image watermarking schemes are widely categorized in to spatial and frequency approaches based on how watermarks are attached to the carrier images (Singh et al., 2017). Since spatial domain techniques attach digital watermark contents directly to the carrier image bits, these methods suffer severely when attacked in terms of watermark inversion and other potential attacks. Hence many frequency domains based algorithms (Wang et al., 2020; Hussain, Razak & Varghese, 2019) are developed using the frequency transformations such as DCT, Radon transform, DFT, Hadamard transform, discrete wavelet transform (DWT), Z-transform, Arnold transform and SVD for mixing carrier and watermark frequencies (Hussain, Razak & Varghese, 2019). Separating different frequencies by these frequency transformations help frequency domain methods to correctly identify and mix appropriate frequency spectrum for attaching watermark contents so that the resultant watermarked image shows better robustness against momentous potential attacks (Hussain, Razak & Varghese, 2019; Nematollahi, Vorakulpipat & Rosales, 2017).

Qin et al. (2017) proposed a least significant bit (LSB) based block-wise tampering localization and pixel-wise recovery for watermarking. But the algorithm is computationally complex and suffers severely with potential attacks since LSBs are the most sensitive bits to potential attacks. Makbol et al. (2017) addressed false positive problem (FPP) by incorporating integer wavelet transform (IWT) and secret keys. However, the algorithm uses computationally inefficient multi-objective ant colony optimization technique for optimizing its parameters. Lei et al. (2018) introduced a watermarking algorithm incorporating quantization index modulation (QIM) to address scaling based potential attacks. Roy & Pal (2018) incorporated redundant discrete wavelet transform (RDWT) with SVD to propose color image watermarking algorithm but the reconstructed watermarked image suffers blocky and patchy effects due to non-overlapping block based processing. Su & Chen (2018) attached watermark components in spatial domain. Liu, Pan & Song (2017) combined fractal coding and DCT methods for enforcing security in their DCT based watermark scheme but the algorithm suffers the limitations of DCT in protecting watermark contents from geometrical potential attacks.

In many frequency domain methods (Garg & Rama Kishore, 2020; Dixit & Dixit, 2017; Kang et al., 2018; Yadav, Kumar & Kumar, 2018; Li et al., 2018; Kaur, Gupta & Singh, 2019; Ali et al., 2015; Zheng et al., 2018; Nguyen, Chang & Yang, 2016), SVD is used in combination with other frequency transformations to provide better quality watermarked images with high robustness towards external potential attacks due to SVD’s simplicity, compactness and geometrical invariance properties (Shih, 2017; Nematollahi, Vorakulpipat & Rosales, 2017; Dixit & Dixit, 2017). Kang et al. (2018) introduced a watermarking algorithm in DCT, SVD and DWT domains by attaching watermark components in specific middle frequencies. However, the algorithm failed in reproducing valuable watermarks when potential attacks affect middle frequency components. Yadav, Kumar & Kumar (2018) attached watermark components in the third level frequency components of DWT. Since this scheme attaches watermark components only in low pass frequencies of first and second levels of DWT, the algorithm could not provide better robustness against attacks that corrupt low frequencies. Li et al. (2018) incorporated Hadamard transform and Schur decomposition. Kaur, Gupta & Singh (2019) attached watermark singular values in the singular value contents of DWT coefficients of the HH components of carrier image. Ali et al. (2015) incorporated artificial bee colony algorithm for selecting suitable non-overlapping blocks for attaching watermark components in wavelet domain. Zheng et al. (2018) used convolution neural network (CNN) to attach watermarks in DWT and SVD domains. However, the algorithm is computationally inefficient due to CNN optimization process with many hidden layers. Nguyen, Chang & Yang (2016) attached the watermark in second level low frequency area of DWT coefficients. Mokashi et al. (2022) used DWT-DCT-SVD transforms to attach watermark images. But the algorithm fails to retrieve correct watermark when potential attacks affect low-frequency sub-bands. These limitations of embedding single copy watermark in specific frequencies, paved way for later algorithms (Emir & Eskicioglu, 2004; Alexander, Scott & Ahmet, 2006; Run et al., 2012; Justin et al., 2016) to attach more copies of watermarks in various carrier frequencies. Hence the algorithms that embed watermark replicas in various carrier frequencies show improved robustness against external potential attacks as these algorithms have more possibility for protecting some watermark replicas although the potential attacks may damage other replicas attached in other carrier image frequencies.

Exploiting the combined merits of SVD and DWT transforms, Emir & Eskicioglu (2004) attached four copies of watermark singular values in the Haar DWT sub-bands. This algorithm produces flaws in watermarked images since it fails to spread the frequencies by applying frequency transformation to the watermark contents before attaching it to the carrier frequencies. Also the problems of DWT in handling JPEG and geometrical attacks produce distortions in its extracted watermarks. Alexander, Scott & Ahmet (2006) attempted to address these limitations by incorporating DCT with SVD, but the algorithm could not provide ample robustness due to the limitations of DCT in handling specific potential attacks. Run et al. (2012) attached principal components of watermarks with the carrier singular values but the algorithm has numerous flaws due to the sensitiveness of principal components towards potential attacks. As an improvement of Alexander, Scott & Ahmet (2006) and Emir & Eskicioglu (2004) algorithms, our previous work (Justin et al., 2016) incorporated DFT and SVD transforms in its watermarking scheme. The algorithm embedded four replicas of watermark singular value components respectively to the singular values corresponding to all the four frequency sub-images. Though this method performs better with majority of potential attacks, but could not provide stability in its performance to provide valid watermarks for all attacks including noise and cropping based attacks. Although these algorithms address some problems of image watermarking, could not simultaneously address all requirements of image watermarking such as capacity, imperceptibility, computational efficiency and robustness against all potential attacks (Shih, 2017; Nematollahi, Vorakulpipat & Rosales, 2017).

In the domain of watermarking, the watermarking techniques incorporating DWT shows better multi-resolution analysis but fails in protecting watermark contents when the carrier image is attacked with JPEG and noise (Poljicak, Mandic & Agic, 2011; Justin et al., 2015). Since DCT is highly used in JPEG image compression standards, it shows good robustness when the watermarked image is subject to lossy compression or in the case of other practical applications on the internet. The watermark contents attached in high frequency bands are damaged as good amount of high frequency coefficients are quantized to zero during JPEG compression. Though DCT show resistance against most of the geometrical attacks, it is highly sensitive to noise and filtering. Since there is a sudden compaction of energy in low frequency area in the top-left pixel of the block of DCT, it is hard to select the suitable middle frequency components of the carrier image for attaching the watermark components. DFT has strong energy compaction with rotation, scaling and translation invariance properties but could not offer comparative resistance in case of noise and cropping based attacks (Poljicak, Mandic & Agic, 2011). It is observed that these transforms though could individually offer better resistance towards some types of potential attacks, fails severely to resist other types of potential attacks (Justin et al., 2016). SVD is a matrix factorization method that factorizes given matrix to two orthonormal matrices and a diagonal singular value matrix with positive real numbers. Even if there are larger changes in the singular value matrix it will not produce much deviation in the reconstructed image. Since singular values possess inherent algebraic advantages and have very good stability, SVD shows good resistance towards noise and cropping based watermark attacks. Considering these advantages and demerits of different transformations, the article presents a new non-blind watermarking algorithm by utilizing the merits of DFT, DCT and SVD to provide stable robustness and imperceptibility qualities.

## Materials and Methods

### New non-blind DFT-DCT-SVD watermarking algorithm

**Figure 1:**
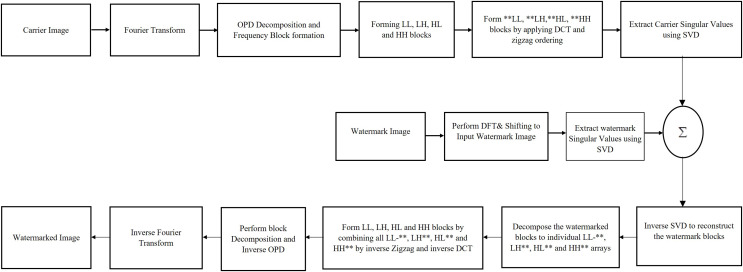
Sequential flow diagram of new scheme.

The new watermarking algorithm exploits advantages of DFT, DCT and SVD transforms while attaching watermark in the carrier image. To achieve improved robustness against external potential attacks, the new method embeds watermark replicas in various carrier frequencies. By embedding watermark replicas to all frequencies of the cover image, the new method provides more chances for protecting some watermark replicas although the potential attacks may damage other replicas. The sequential flow diagram of new scheme is shown in Fig. 1.

### Embedding the watermark

The algorithm begins by transforming carrier image to frequency domain by applying Fourier transform and shifting its origin to the center. The resultant image is then divided in to four frequency blocks depending on its circular distance from the direct current (DC) coefficient using OPD algorithm (Justin et al., 2016). DCT is applied to these four frequency bands and the resultant image is ordered in zigzag fashion to form four individual frequency arrays. These individual arrays are again equally divided into four frequency spectrums, the extremely high (B4), high (B3), medium (B2) and very low frequency (B1) arrays. Similar frequency components of these arrays are combined for forming final four frequency blocks and SVD transform is individually applied to these four carrier image frequency blocks. In parallel to these operations, the watermark image is also applied with Fourier transform followed by SVD and the copies of watermark singular values are attached with the singular values of cover image frequency blocks.

If *C* and *X* denote cover and watermark images with dimensions $m \times n$ and $\displaystyle{m \over 2} \times \displaystyle{n \over 2}$ respectively, then new watermarking scheme is explained in the subsequent steps.

**Step 1:** Fourier transform provides energy compaction with strong tolerance towards translation and rotation (Setiadi, 2021) and hence the algorithm applies Fourier transform on *C* and *X* to form frequency domain images, *F* and *W* respectively.

(1) $${F_{u,v}} = \displaystyle{1 \over {mn}}\sum\limits_{{i } = 0}^{{m } - 1} {\sum\limits_{{j } = 0}^{n - 1} {{C_{ij}}{e^{ - 2\pi j\left( {\displaystyle{{ui} \over m} + \displaystyle{{vj} \over n}} \right)}}} }$$

(2) $${W_{_{x,y}}} = \displaystyle{4 \over {mn}}\sum\limits_{i = 0}^{m/2 - 1} {\sum\limits_{{j } = 0}^{n/2 - 1} {{X_{ij}}{e^{ - 4\pi j\left( {\displaystyle{{xi} \over m} + \displaystyle{{vj} \over n}} \right)}}} }$$

**Figure 2:**
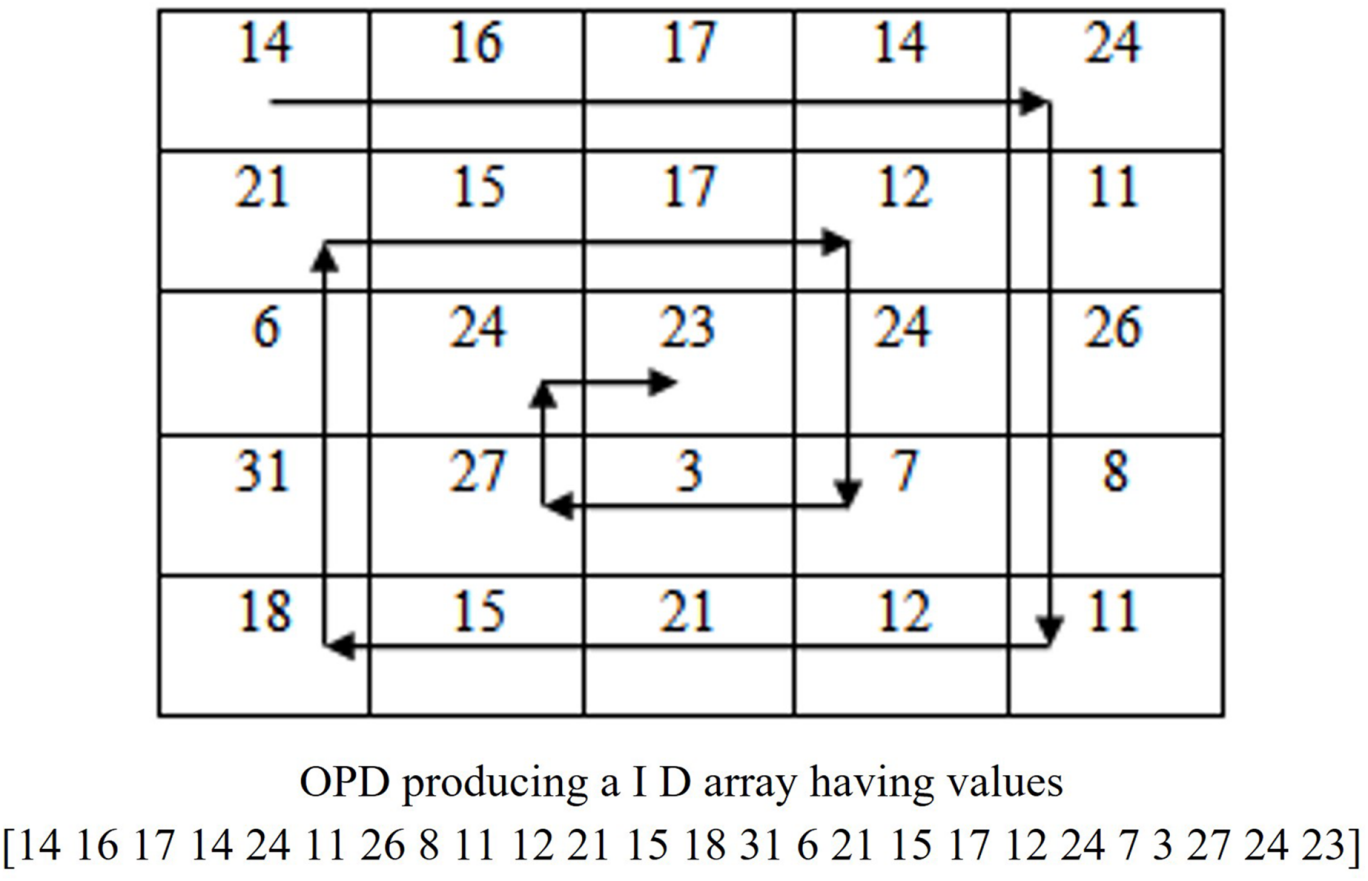
Illustration of OPD traversal with example.

**Step 2:** For utilizing the advantages of attaching watermark replicas to different frequency levels, the algorithm converts *F* to one dimensional frequency array using OPD. OPD algorithm performs a circular sweep of Fourier spectrum starting from the upper left corner (high frequency components) to the DC coefficient (lowest frequency components) located at the center of *F*. The OPD track all the frequencies of DFT spectrum in the high to low frequency order. Since the frequencies in DFT image, *F* vary from low to high frequencies in proportion to its positional distance from the DC component at the center position, the output of OPD is a 1D frequency array whose values starts at the highest frequency and ends at the DC coefficient. If this array is divided in to four equal parts, it is easy to separate the frequency components. These sub-bands are reshaped to 2D shape to form ultra high, very high, high and low frequency sub-band images. The singular value components of these sub-band images are then used for attaching multiple copies of watermark components. The OPD algorithm with its demonstrating example is given in Fig. 2. Let B4, B3, B2 and B1 denote extremely high, high, medium and very low frequency arrays of equal size $1 \times \displaystyle{{mn} \over 4}$. The OPD operation is defined as

(3) $$[HH\, HL\, LH\, LL] = OPD\left( F \right).$$

Pictorial illustration of OPD and inverse OPD are given in Fig. 2 and the details of the algorithm with pseudo codes are found in our older work (Justin et al., 2016). These B4, B3, B2 and B1 arrays are reshaped into $\displaystyle{m \over 2} \times \displaystyle{n \over 2}$ blocks for further processing and the corresponding frequency blocks formed are denoted as *B*_*1*_, *B*_*2*_, *B*_*3*_ and *B*_*4*_ blocks.

**Step 3:** To provide better robustness towards JPEG compression based external attacks, DCT is applied on *B*_*1*_, *B*_*2*_, *B*_*3*_ and *B*_*4*_ frequency blocks as a second level frequency decomposition to obtain the frequency block $F{D_k}$ for all the four blocks $k = 1,2,..{4}$.

Since the frequencies in the DCT image, $F{D_k}$ vary from low to high in proportion to its diagonal distance from the DC component at position $\left( {0,{0}} \right)$, each $F{D_k}$ is ordered in zigzag manner to convert $FD$ to one dimensional frequency array where values of $F{D_k}$ starts at low frequency and ends at high frequency coefficient. When these individual arrays are divided into four equal parts (totally 16 for all four blocks), it is easy to separate the frequency components into the second level of B4, B3, B2 and B1 components. From all these individual arrays, the B4, B3, B2 and B1 counterparts are individually collected and four frequency arrays with equal sizes of $1 \times \displaystyle{{MN} \over 4}$. Further, if the frequency components which belong to $F{D_1}$ are denoted as B1B1, B1B2, B1B3 and B1B4, those which belong to $F{D_2}$ are denoted as B2B1, B2B2, B2B3, B2B4, those which belong to $F{D_3}$ are denoted as B3B1, B3B2, B3B3, B3B4 and those which belong to $F{D_4}$ are denoted as B4B1, B4B2, B4B3, B4B4, then these combined frequency arrays are formed by combining and reshaping (B1B1 B3B1 B2B1 B4B1), (B1B2 B3B2 B2B2 B4B2), (B1B3 B3B3 B2B3 B4B3) and (B1B4 B3B4 B2B4 B4B4) arrays to individual blocks ${D_k}$ $\forall k = 1,2,..{4}$ of size $\displaystyle{M \over 2} \times \displaystyle{N \over 2}$.

**Step 4:** Since external modification of the carrier image doesn’t affect the singular values much, SVD of individual frequency blocks ${D_k}$ $\forall k = 1,2,..{4}$ are then determined for attaching the copy of watermark singular values with the carrier frequency block singular values. The SVD operation on carrier and watermark frequency blocks are defined respectively as

(4) $${D_k} = {U_k}{S_k}V_k^{^T}\quad \forall {1 } \le k \le 4$$

(5) $$W = \mathop {{\rm{ }}U}\limits^X \mathop {{\rm{ }}S}\limits^X \mathop V\limits^{{X^T}} .$$

where *T* performs transpose, ${U_k}$, ${V_k}$, $\mathop U\limits^X$, $\mathop V\limits^X$ are orthonormal matrices while ${S_k}$ and $\mathop S\limits^X$ are singular value matrices. The diagonal elements of ${S_k}$ and $\mathop S\limits^X$ are called singular values of cover and watermark blocks, respectively.

**Step 5:** Embedding watermark content replicas at different frequency bands of cover image help protect watermark contents from external attacks affecting specific frequencies and hence the watermark embedding process is done by adding the replicas of watermark singular value matrix $\mathop S\limits^X$ to the singular value matrices of carrier frequency block as

(6) $$S_k^* = {S_k} + {\eta _k}\mathop S\limits^X \quad \forall {1 } \le k \le 4.$$

Here ${\eta _k}$ and $S_k^*$ represent embedding strength parameter and singular values corresponding to carrier image respectively of the *k*^*th*^ block. The embedding intensity ${\eta _k}$ controls the watermark contents in cover image and it is set according to the trust on the communication media and the quality of watermarked image for specific applications.

**Step 6:** Once the watermark contents are attached with cover image frequencies, the algorithm starts the inverse process to reproduce the watermarked image. The watermarked frequency blocks ${\mathop D\limits^* _k}$ $\forall k = 1,2,..{4}$ are determined by performing inverse SVD as

(7) $${\mathop D^*}_k = {U_k}{\mathop S\limits^* }_kV_k^{^T}\quad \forall {1 } \le k \le 4.$$

**Step 7:** These watermarked frequency blocks ${\mathop D\limits^* }_k$ $\forall k = 1,2,..{4}$ are then reshaped back to size of $1 \times \displaystyle{{MN} \over 4}$ and are divided in to four equal parts (totally 16 inclusive of all four blocks). The reordered and combined frequency arrays are formed by rearranging (B1B1 B3B1 B2B1 B4B1), (B1B2 B3B2 B2B2 B4B2), (B1B3 B3B3 B2B3 B4B3) and (B1B4 B3B4 B2B4 B4B4) arrays of $\mathop {{D_k}}\limits^*$ $\forall k = 1,2,..{4}$ to ${\mathop {FD}\limits^* }_k$ such that ${\mathop {FD}\limits^* }_1$ is (B1B1, B1B2, B1B3 B1B4), ${\mathop {FD}\limits^* }_2$ is (B2B1, B2B2, B2B3, B2B4), ${\mathop {FD}\limits^* }_3$ (B3B1, B3B2, B3B3, B3B4) and ${\mathop {FD}\limits^* }_4$ (B4B1, B4B2, B4B3, B4B4) arrays.

**Step 8:** Since these rearranged arrays are in DCT and zigzag ordered form, inverse zigzag operation followed by inverse DCT are applied to these individual arrays to find the next level of processing to reconstruct the watermarked image. Let ${\mathop B\limits^* }_k$ $\forall k = 1,2,..{4}$ denote the frequency block after applying inverse zigzag and the inverse DCT operations.

**Step 9:** The frequency blocks, ${\mathop B\limits^* }_k$ $\forall k = 1,2,..{4}$ are individually reshaped into four arrays to form B1, B2, B3 and B4 arrays to form the combined array (B4, B3, B2, B1). The inverse OPD algorithm is then applied to reconstruct back the Fourier transform of the watermarked image as

(8) $$\mathop F\limits^* { = IOPD}\left( {\left[ {B4,{ B3, B2, B1}} \right]} \right).$$

**Step 10:** As the final stage, Inverse Fourier Transform is performed on $\mathop F\limits^*$ for regenerating the watermarked image, $\mathop C\limits^*$ as

(9) $${\mathop C\limits^* _{_{ij}}} = \sum\limits_{u = 0}^{M - 1} {\sum\limits_{{ v } = 0}^{N - 1} {{{\mathop F\limits^* }_{uv}}\ {e^{2\pi j\left( {\displaystyle{{ui} \over M} + \displaystyle{{vj} \over N}} \right)}}} }.$$

The watermark components $\mathop S\limits^X$ attached inside watermarked image $\mathop C\limits^*$ is utilized as a meta-data to identify the owner, authentication and uprightness of the host images. The scheme has better energy compaction, geometrical invariance and other resistance properties towards external attacks due to the collective advantages of different transforms. Also the algorithm is capable producing better quality watermarked outputs with high visual qualities due to better energy spreading capabilities of different transforms.

### Extraction of watermark

This sub-section explains the operations to be performed for extracting the watermark from carrier image. The algorithm performs the same way as embedding process but extracts the watermark contents from watermarked singular values. If $\tilde {C}$ denotes watermarked image received for authentication, the watermark extraction procedure is detailed through following steps:

**Step 1:** The watermark extraction algorithm starts by applying Fourier transformation on the watermarked image, $\tilde {C}$ as in (1) to produce $\tilde F$.

**Step 2:** As used in watermark embedding process, OPD is applied for converting $\tilde F$ to one dimensional frequency array as

(10) $$\left[ {B4,{ B3, B2, B1}} \right]{ = OPD}\left( {\tilde F } \right)$$

The B4, B3, B2 and B1 arrays are reshaped into $\displaystyle{m \over 2} \times \displaystyle{n \over 2}$ blocks for further processing and these frequency blocks are denoted as ${\tilde B _1}$, ${\tilde B _2}$, ${\tilde B _3}$, and ${\tilde B _4}$.

**Step 3:** DCT is applied to ${\tilde B _1}$, ${\tilde B _2}$, ${\tilde B _3}$, and ${\tilde B _4}$ frequency blocks for obtaining second level frequency decomposition, $\tilde {FD}$. Each ${\tilde {FD} _k}$ is ordered in zigzag fashion for converting $\tilde {FD}$ to one dimensional frequency array whose values starts from low frequency coefficients and ends at high frequency coefficients, *i.e*., B1B1, B1B2, B1B3 and B1B4 belong to ${\tilde {FD} _1}$, B2B1, B2B2, B2B3, B2B4 belong to ${ \tilde {FD} _2}$, B3B1, B3B2, B3B3, B3B4 belong to ${\tilde{FD} _3}$ and B4B1, B4B2, B4B3, B4B4 belong to ${\tilde {FD} _4}$. Further, these arrays are reshaped to individual blocks ${\tilde D _k}$ $\forall k = 1,2,..{4}$ with sizes $\displaystyle{m \over 2} \times \displaystyle{n \over 2}$.

**Step 4:** Same as step 4 of watermark embedding process, SVD on individual frequency blocks, ${\tilde D _k}$ $\forall k = 1,2,..{4}$ are performed for extracting the watermark singular value replicas from carrier block singular values as

(11) $$\tilde {{D_k}} = {\tilde {U} _k}{\tilde{ S} _k}\tilde {{{ V}_{k}}^{^T}}\quad \forall { 1 } \le k \le 4.$$

**Step 5:** The watermark singular value copies $\mathop {{S_k}}\limits^X$ are then extracted from the watermarked frequency block singular value matrices as

(12) $$\tilde { {{S_k}} }^X = \displaystyle{{\left( {{{\tilde {S}}_k} - {S_k}} \right)} \over {{\eta _k}}}$$

where ${\eta _k}$ and $S_k^*$ are the embedding strength parameter and hast image singular values of *k*^*th*^ block, respectively.

**Step 6:** The watermarked blocks are constructed by applying inverse SVD to respective watermark singular values

(13) $${\mathop W\limits^\~ _k} = \mathop {{\rm{ }}U}\limits^X \mathop {{\rm{ }}S}\limits^{X\~} \mathop {{V^T}}\limits^X .$$

**Step 7:** As the final step, the algorithm applies inverse Fourier transform on ${\tilde W_k}$ and the watermark replicas $\tilde{ X _k}$ are extracted as

(14) $${\tilde X _k}_{_{ij}} = \sum\limits_{u = 0}^{\textstyle{m \over 2} - 1} {\sum\limits_{{v } = 0}^{\textstyle{n \over 2} - 1} {{{\tilde W }_{u,v}}\ {e^{4\pi j\left( {\displaystyle{{ui} \over m} + \displaystyle{{vj} \over n}} \right)}}} }.$$

**Figure 3:**
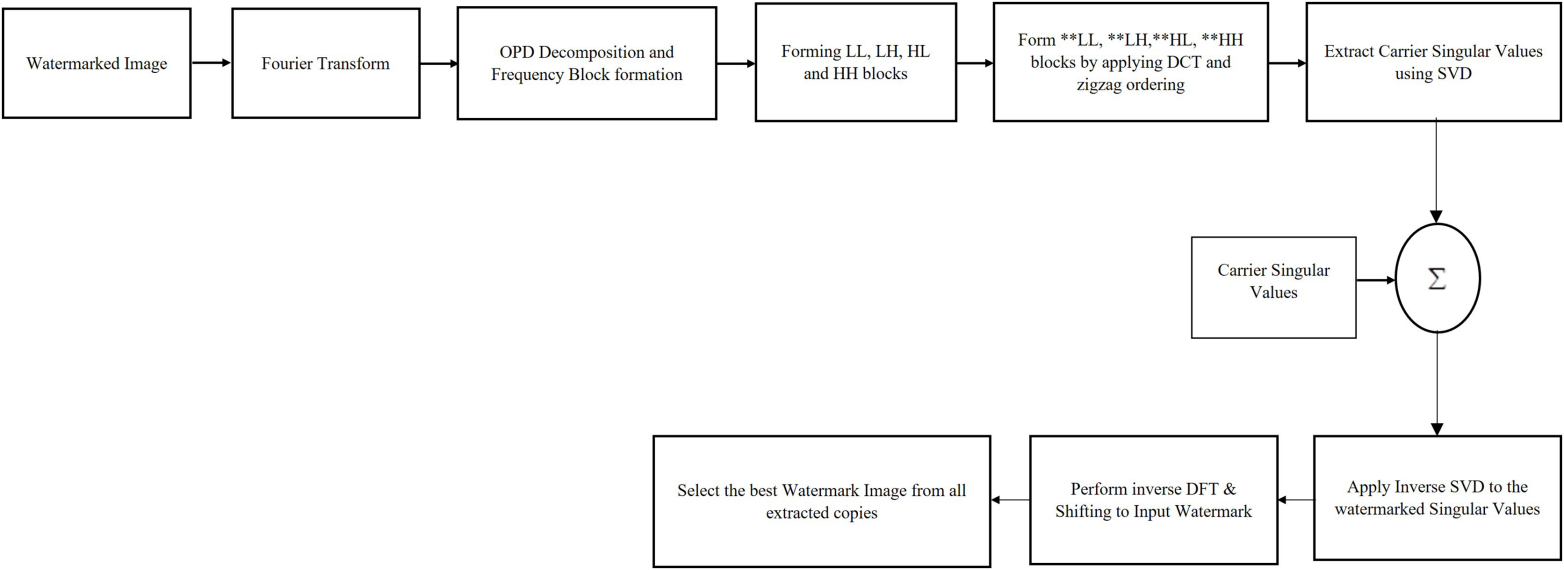
Sequential flow diagram of watermark extraction process. Test images are from https://ccia.ugr.es/cvg/CG/base.htm and https://in.mathworks.com/help/images/ open databases.

Since the scheme exploit the merits of DFT, DCT and SVD transforms to protect watermark contents from wide levels of potential attacks through its watermark embedding and extraction schemes, it consistently produces better quality watermarked images with better robustness. Since it attaches singular value replicas to all frequency contents of cover image frequencies, the algorithm has better provision for protecting watermarks from various sets of attacks that affect specific frequency bands of cover images. Also, as the algorithm apply Fourier transform to the watermark before it is attached with cover image contents, it ensures better frequency spreading of watermark contents in cover image contents ensuring high quality watermarked images with better resistance towards external attacks. The sequential flow diagram of extracting watermark is given in Fig. 3.

## Results

The empirical analysis of the watermarking method based on imperceptibility and robustness capabilities is made with a large set of images from which rice, man, circles, Barbara, cameraman, boats, baboon, peppers, bridge and different logo images are presented in this article for demarcating the subjective and objective performances of different algorithms. To perform effective comparative study of various watermarking schemes with subjective and objective metrics, the payload of watermark content in cover image needs to be same for these algorithms. Aiming at these requirements, the algorithms that attach similar amount of data with similar multiple replicas are being considered in this study for analysis. Comparative algorithms used in the study to analyse the effectiveness of the new scheme are DCT based Run et al. (2012), DWT based Run et al. (2012), Mokashi et al. (2022), Emir & Eskicioglu (2004), Alexander, Scott & Ahmet (2006), and Justin et al. (2016) algorithms. Mokashi et al. (2022) algorithm attaches watermarked watermarks in DCT domain of LL sub-bands. However, for this experimental comparison, the watermarks used are not watermarked with signature of the owner.

Imperceptibility measures the similarity of watermarked images with its original counterpart while robustness makes the tolerance of watermarked images to external potential attacks in preserving watermark contents. The imperceptibility analysis of the new algorithm is made with peak signal to noise ratio (PSNR) (Setiadi, 2021), mean structural similarity index measure (MSSIM) (Setiadi, 2021; Wang et al., 2004) and feature similarity index measure (FSIM) (Zhang et al., 2011). PSNR is determined as

(15) $$PSNR = 10{log _{10}}\left( {\displaystyle{{{{\left( {255} \right)}^2}} \over {MSE}}} \right)\left( {{ dB}} \right).$$

Here, MSE is the mean square error of $\mathop C\limits^*$ with *C*. SSIM provides human perception-based structural similarity analysis between *C* and $\mathop C\limits^*$ (Wang et al., 2004). MSSIM is given by

(16) $$MSSIM = \displaystyle{1 \over k}\sum\limits_{b = 1}^k {\displaystyle{{\left( {2{\mu _{_{{C_{_b}}}}}{\mu _{C_{_{_b}}^*}} + {c_{_1}}} \right)\left( {2{\sigma _{_{{C_{_b}}C_{_{_b}}^*}}} + {c_{_2}}} \right)} \over {\left( {{\mu _{_{{C_{_b}}}^2 + \mu _{C_{_{_b}}^*}^2 + }}{c_1}} \right)\left( {{\sigma _{_{{C_{_b}}}^2 + \sigma _{C_{_{_b}}^*}^2 + }}{c_{_2}}} \right)}}}.$$

Here, ${C_{_b}}$ and $$C_{_b}^{*}$$ respectively represents image areas of *b*^*th*^ window of *C* and $$\mathop C\limits^* $$. Also $\mu$ and $\sigma$ denote the mean and standard deviation while *c*_*1*_ and *c*_*2*_ are constants. FSIM provides human perception based low-level feature comparison measure by extracting phase congruency (PC) and gradient magnitude (GM) of comparative images. If $\Omega$ is the set of entire row and column positions of *C* and $\mathop C\limits^*$, FSIM is determined by

(17) $$FSIM\left( {C,{C^*}} \right) = \displaystyle{{\sum\limits_{b \in \Omega } {SL\left( b \right) \times P{C_m}\left( b \right)} } \over {\sum\limits_{b \in \Omega } {P{C_m}\left( b \right)} }}.$$

Here, $SL\left( b \right)$ is the local similarity map by phase congruency (PC) and gradient magnitude (GM) while $P{C_m}\left( b \right)$ represents maximum of phase congruency of *C* and $\mathop C\limits^*$. Detailed explanation on FSIM calculations can be found in Zhang et al. (2011).

For maintaining consistency in comparative analysis of various schemes, the embedding strength parameter ${\eta _k}$ $\forall k = 1,2,..{4}$ is set to 0.1 for all algorithms.

Robustness of the new scheme is analyzed with other algorithms using bit error rate (BER) in percentage and normalized cross correlation (NCC) values of attached and extracted watermark images and the Pearson’s correlation coefficient (PCC) values of attached and extracted singular values against a variety of external attacks, from which Gaussian and impulse noises, geometrical transformations, filtering, histogram equalization, JPEG compression, resizing, unsharp masking, contrast stretching, flipping and pixelate attacks are used in this article to illustrate the analysis. Bit error rate (BER) (Mokhnache, Bekkouche & Chikouche, 2018) represents the ratio of corrupted bits to total available bits. The BER of extracted watermark, $\tilde X$ from attached watermark, *X* in percentage is determined as

(18) $$BER = \displaystyle{{\sum\limits_{i = 0}^{M - 1} {\sum\limits_{j = 0}^{N - 1} {X\left( {i,j} \right) \otimes \tilde X \left( {i,j} \right)} } } \over {M \times N}} \times 100\left( \rm \% \right).$$

Here $\otimes$ represents the XOR operation. An effective watermarking algorithm should produce low BER in case of potential attacks affected watermarked images. The normalized cross correlation (NCC) (Xie & Qin, 2010) measures the resemblance of attached and extracted watermarks and is defined by

(19) $$NCC = \displaystyle{{\sum\limits_{i = 0}^{M - 1} {\sum\limits_{j = 0}^{N - 1} {X\left( {i,j} \right) \times \tilde X \left( {i,j} \right)} } } \over {\sqrt {\sum\limits_{i = 0}^{M - 1} {\sum\limits_{j = 0}^{N - 1} {{{\left( {X\left( {i,j} \right)} \right)}^2}} } } \times \sqrt {\sum\limits_{i = 0}^{M - 1} {\sum\limits_{j = 0}^{N - 1} {{{\left( {\tilde X \left( {i,j} \right)} \right)}^2}} } } }}.$$

High values of NCC near one indicates that the output watermark of algorithm has high s resemblance with the original watermark. PCC is used for measuring the linear relationship of original singular values $\mathop {S}\limits^{X}$ with the extracted singular values $\mathop {\tilde {{S_k}} }\limits^X$ from watermarked image impinged with different attacks. PCC calculation can be expressed by

(20) $$PCC = {{\sum \left( {\mathop {{\rm{ }}S}\limits^X  - {\mu _{\mathop {{\rm{ }}S}\limits^X }}} \right)\left( {\mathop {S_k^\sim}\limits^X X - {\mu _{{{\widetilde {{S_k}}}^X}}}} \right)} \over {\sqrt {\sum {\left( {\mathop S\limits^X  - {\mu _{\mathop {{\rm{ }}S}\limits^X }}} \right)} } \sum {{\left( {\mathop {S_k^\sim}\limits^X  - {\mu _{{{\widetilde {{S_k}}}^X}}}} \right)}^2}}}.$$

Here, ${\mu _{{S}_{k}^{*}}}$ and ${\mu _{\mathop {S_k^*}\limits^X }}$ respectively denote the mean of $\mathop S\limits^X$ and $\mathop {\tilde {{S_k}} }\limits^X$. The result of PCC varies from +1 to −1, where +1 indicates positive linear correlation, 0 indicates non-correlation, and −1 indicates negative correlation. From all extracted watermark replicas ${\tilde X _k}$ $\forall k = 1,2,..{4}$, the best replica in terms of PCC values of its singular values are used for analyzing the performance of all algorithms used in the study.

In order to identify the best combination of transformations from DWT, DCT and DFT that shows better perceptibility qualities, experiments are conducted on DWT-DCT-SVD, DWT-DFT-SVD and DCT- DFT-SVD combinations with 15 carrier and watermark images and the perceptibility quality results of watermarked images from these methods are compared based on PSNR, MSSIM and FSIM values as presented in Table 1. Table 1 show that DFT-DCT-SVD scheme produces better PSNR, MSSIM and FSIM values than DWT-DCT-SVD and DWT-DFT-SVD based algorithms except for the MSSIM values of Barbara-Cameraman and FSIM values of Bridge- Logo-4 carrier-watermark image combinations. The robustness comparison of DWT-DCT-SVD, DWT-DFT-SVD and DFT- DCT-SVD based methods are performed with BER and NCC values of attached and extracted watermark images and the PCC values of attached and extracted singular values against different image processing attacks. Average BER, NCC and PCC values produced by DWT-DCT-SVD, DWT-DFT-SVD and DFT- DCT-SVD from 15 watermark images are presented in Table 2. From Table 2, it is vivid that the DFT-DCT-SVD algorithm produces better BER, NCC and PCC values than DWT-DCT-SVD and DWT-DFT-SVD based algorithms except for rescaling/multi-resolution based potential attacks. By analyzing the quantitative results of DWT-DCT-SVD, DWT-DFT-SVD and DFT-DCT-SVD algorithms from Tables 1 and 2, it can be identified that DFT- DCT-SVD algorithm shows better imperceptibility and robustness qualities than DWT-DCT-SVD and DWT-DFT-SVD algorithms and hence, we use DFT- DCT-SVD algorithm as the proposed method to compare other prominent algorithms used in this comparative study.

**Table 1:** PSNR, SSIM and FSIM analysis of watermarked images of different algorithms with embedding strength parameter $\eta_{k}$ = 0.1.

Carrier image	Watermark image	DWT-DCT-SVD			DWT-DFT-SVD			Proposed scheme (DCT- DFT-SVD)		
		PSNR	MSSIM	FSIM	PSNR	MSSIM	FSIM	PSNR	MSSIM	FSIM
Lena	Rice	34.477	0.957	0.992	33.780	0.967	0.953	34.825	0.987	0.993
Man	Circles	33.893	0.944	0.966	34.931	0.963	0.947	34.985	0.983	0.983
Barbara	Cameraman	32.336	0.988	0.966	33.683	0.988	0.986	33.721	0.986	0.992
Boats	Logo-1	28.539	0.946	0.925	28.539	0.936	0.916	29.728	0.975	0.954
Baboon	Logo-2	31.668	0.972	0.958	31.040	0.962	0.949	31.754	0.992	0.978
Peppers	Logo-3	30.034	0.962	0.919	29.133	0.972	0.900	30.752	0.974	0.938
Bridge	Logo-4	28.963	0.947	0.962	28.086	0.956	0.974	29.256	0.986	0.971
Average of 15 sets of images		32.215	0.973	0.937	31.813	0.983	0.962	33.045	0.989	0.972

**Table 2:** Average robustness analysis of extracted 15 watermark images from various cover images impinged with different external attacks by various schemes.

External attacks	DWT-DCT-SVD			DWT-DFT-SVD			Proposed scheme (DCT- DFT-SVD)		
	BER	NCC	PCC	BER	NCC	PCC	BER	NCC	PCC
Gaussian noise with noise ratio 0.001	37.105	0.912	0.930	34.887	0.949	0.933	34.645	0.974	0.978
Gaussian filtering 3 × 3	36.875	0.902	0.948	36.934	0.956	0.967	36.746	0.994	0.994
Gaussian filtering 5 × 5	36.824	0.963	0.926	36.927	0.907	0.992	36.776	0.994	0.993
Gaussian filtering 7 × 7	36.871	0.931	0.922	36.989	0.897	0.952	36.776	0.994	0.997
Histogram equalization	37.307	0.920	0.941	37.478	0.942	0.934	37.292	0.969	0.968
JPEG Compression with 75% quality	26.369	0.946	0.916	26.448	0.890	0.944	26.259	0.983	0.984
JPEG compression with 50% quality	30.061	0.910	0.966	30.064	0.930	0.974	30.055	0.975	0.979
Rotation 10°	37.831	0.899	0.923	37.891	0.922	0.901	37.616	0.931	0.937
Rotation 20°	28.548	0.943	0.908	28.259	0.948	0.954	28.862	0.941	0.947
Image resizing 512–>256	36.496	0.944	0.910	36.130	0.956	0.964	36.565	0.954	0.959
Image resizing 512–>1,024	33.530	0.952	0.925	33.394	0.985	0.952	33.304	0.987	0.989
Unsharp masking	38.229	0.858	0.877	38.538	0.905	0.920	38.171	0.933	0.934
Gamma correction $\gamma=0.8$	36.349	0.937	0.908	36.349	0.910	0.905	36.028	0.966	0.977
Gamma correction $\gamma=0.6$	36.776	0.791	0.841	36.666	0.801	0.858	36.419	0.833	0.870
Impulse noise 1%	36.745	0.869	0.875	36.701	0.861	0.946	36.551	0.954	0.951
Impulse noise 5%	35.530	0.963	0.956	35.220	0.969	0.982	35.206	0.996	0.996
Row flipping	34.610	0.924	0.887	34.796	0.883	0.895	34.496	0.938	0.937
Column flipping	33.819	0.900	0.974	33.971	0.958	0.981	33.674	0.984	0.984
Pixelate with 2 × 2 tiles	35.583	0.931	0.949	35.544	0.937	0.895	35.434	0.991	0.991
Pixelate with 4 × 4 tiles	37.853	0.984	0.912	38.061	0.903	0.941	37.785	0.998	0.991

**Figure 4:**
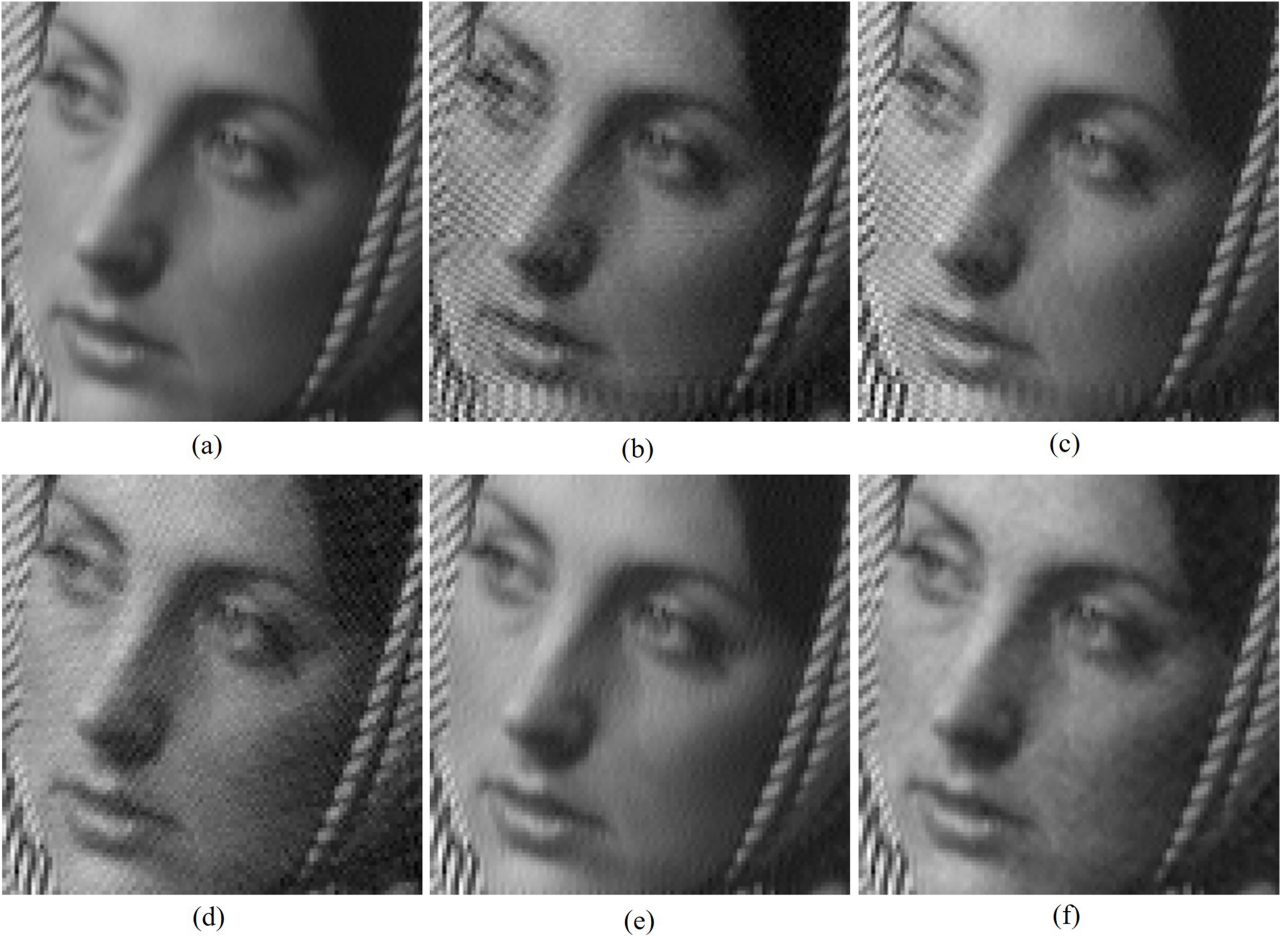
Cropped versions of watermarked Barbara (cover) images produced by various algorithms with the cameraman image as watermark. (A) Original cover image, (B) Run et al. (2012), (C) Emir & Eskicioglu (2004), (D) Alexander, Scott & Ahmet (2006), (E) Justin et al. (2016), (F) proposed scheme. Test images are from https://ccia.ugr.es/cvg/CG/base.htm and https://in.mathworks.com/help/images/ open databases.

Tables 3–5 respectively shows the PSNR, SSIM and FSIM comparison of watermarked outputs by various algorithms and these results are used for analyzing the imperceptibility capabilities of proposed algorithm against Run et al. (2012), DWT based Run et al. (2012), Emir & Eskicioglu (2004), Alexander, Scott & Ahmet (2006) and Justin et al. (2016) methods. Table 3 show that the proposed scheme produces better PSNR values than other comparative methods except for combination between Bridge and Logo-4. From Table 4, it is clear that the new scheme produces better MSSIM values than other methods except for the man and circle combination. Table 5 demarcates that the proposed scheme produces better FSIM values than other methods except for Baboon-Logo-2 and peppers- Logo-3 combinations. Figure 4 shows the cropped versions of watermarked images by comparative algorithms for Barbara (cover) image with the cameraman image as watermark. By analyzing the quantitative values of Tables 3–5 and by the visual analysis on Fig. 4, one can reach that the proposed scheme shows stable performance and it outperforms other algorithms in most of the test cases of imperceptibility analysis.

**Table 3:** PSNR determined from watermarked images of different algorithms with embedding strength parameter $\eta_{k}$ = 0.1.

Carrier image	Watermark image	PSNR values (dB)					
		Run et al. (2012)	Run et al. (2012)	Emir & Eskicioglu (2004)	Alexander, Scott & Ahmet (2006)	Justin et al. (2016)	Proposed scheme
Lena	Rice	26.614	26.440	26.778	26.614	34.777	34.825
Man	Circles	26.662	26.756	26.863	26.662	34.415	34.985
Barbara	Cameraman	25.582	25.435	25.725	25.582	33.413	33.721
Boats	Logo-1	21.555	21.460	21.648	21.555	29.539	29.728
Baboon	Logo-2	23.138	23.019	23.252	23.138	31.293	31.754
Peppers	Logo-3	21.942	21.845	22.125	21.981	29.913	30.752
Bridge	Logo-4	21.446	21.357	21.532	21.446	29.354	29.256

**Table 4:** MSSIM determined from watermarked images of different algorithms with embedding strength parameter $\eta_{k}$ = 0.1.

Carrier image	Watermark image	MSSIM values					
		Run et al. (2012)	Run et al. (2012)	Emir & Eskicioglu (2004)	Alexander, Scott & Ahmet (2006)	Justin et al. (2016)	Proposed scheme
Lena	Rice	0.877	0.991	0.986	0.900	0.987	0.987
Man	Circles	0.875	0.963	0.959	0.919	0.985	0.983
Barbara	Cameraman	0.889	0.991	0.978	0.913	0.985	0.986
Boats	Logo-1	0.820	0.987	0.966	0.850	0.975	0.975
Baboon	Logo-2	0.893	0.992	0.987	0.913	0.992	0.992
Peppers	Logo-3	0.740	0.970	0.969	0.793	0.962	0.974
Bridge	Logo-4	0.858	0.980	0.983	0.899	0.986	0.986

**Table 5:** FSIM determined from watermarked images of different algorithms with embedding strength parameter $\eta_{k}$ = 0.1.

Carrier image	Watermark image	FSIM values					
		Run et al. (2012)	Run et al. (2012)	Emir & Eskicioglu (2004)	Alexander, Scott & Ahmet (2006)	Justin et al. (2016)	Proposed scheme
Lena	Rice	0.926	0.996	0.989	0.943	0.991	0.993
Man	Circles	0.942	0.991	0.981	0.957	0.976	0.983
Barbara	Cameraman	0.934	0.956	0.991	0.946	0.989	0.992
Boats	Logo-1	0.889	0.993	0.948	0.905	0.951	0.954
Baboon	Logo-2	0.939	0.995	0.969	0.949	0.997	0.978
Peppers	Logo-3	0.869	0.985	0.998	0.889	0.990	0.938
Bridge	Logo-4	0.912	0.925	0.965	0.927	0.964	0.971

**Figure 5:**
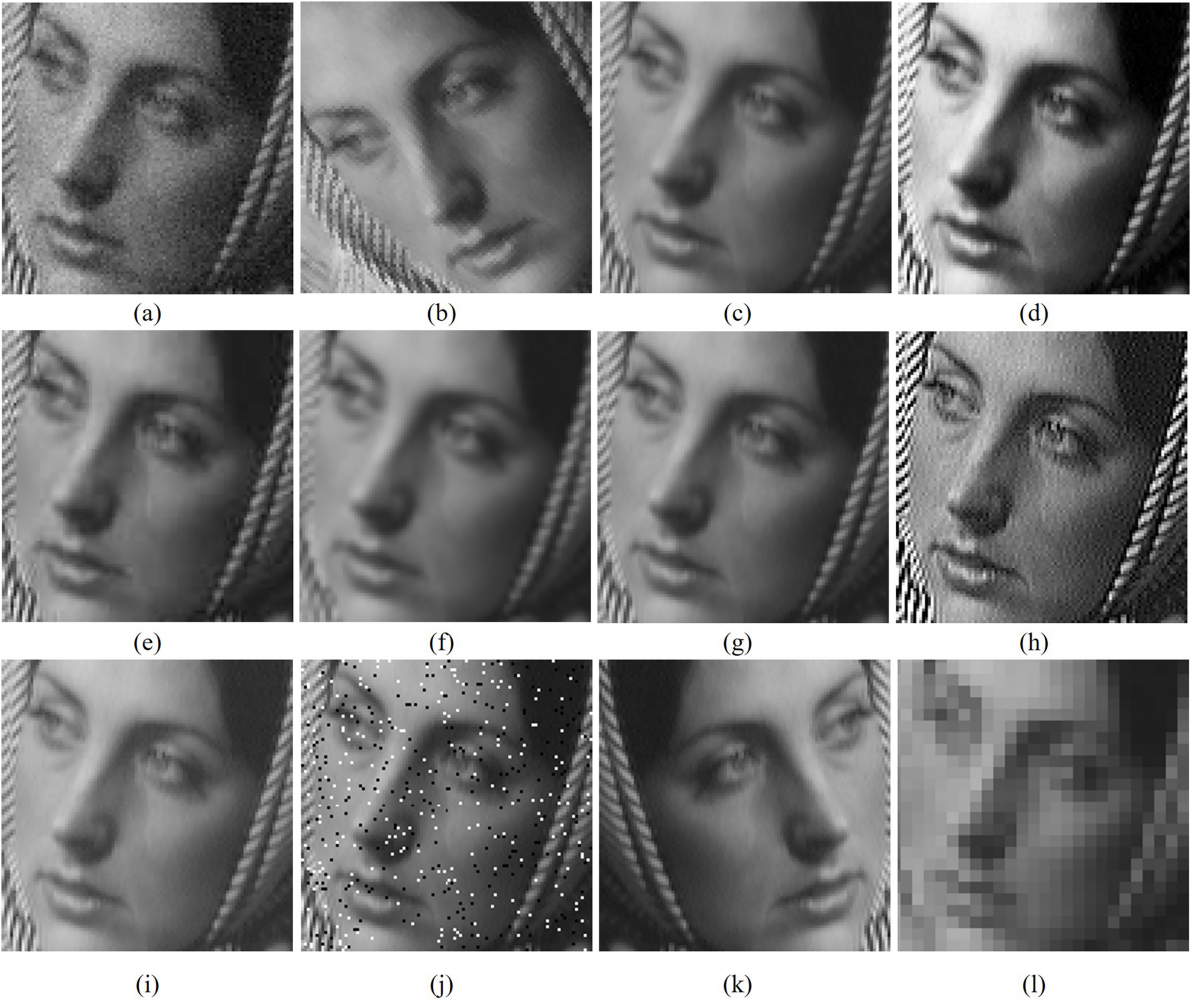
Cropped versions of watermarked Barbara image impinged with various attacks. (A) Gaussian noise with noise ratio 0.001, (B) rotation 45° (C) Gaussian filter 7 × 7, (D) histogram Equalization, (E) jPEG with 50% quality, (F) image Resizing (512-256-512), (G) image Resizing (512-1024-512), (H) unsharp Masking, (I) contrast Stretching, (J) impulse noise with 5% noise, (K) column Flip, (L) pixelate 4 × 4 tiles. Test images are from the https://ccia.ugr.es/cvg/CG/base.htm open database.

Figure 5 shows the cropped versions of watermarked Barbara image impinged with different attacks. Tables 6–8 makes the PCC analysis of watermark images of various schemes from watermarked images impinged with different external attacks. From Table 6, it is clear that Emir & Eskicioglu (2004), Alexander, Scott & Ahmet (2006), Justin et al. (2016) algorithms produce best results for seven, 10 and five potential attacks respectively while the proposed method produces best results for 15 image processing attacks. Table 7 demarcates that Emir & Eskicioglu (2004), Alexander, Scott & Ahmet (2006), Justin et al. (2016) algorithms produce best results for two, nine and five potential attacks respectively while the proposed method produces best results for 17 image processing attacks. Table 8 shows that Emir & Eskicioglu (2004), Alexander, Scott & Ahmet (2006), Justin et al. (2016) algorithms respectively produce best results for five, 12 and four potential attacks while the proposed method produces best results for 15 image processing attacks. Tables 9–11 makes the BER and NCC analysis of watermark images of various schemes from watermarked images impinged with different external attacks.

**Table 6:** PCC analysis of extracted Rice watermark image from Lena cover image impinged with different external attacks by various schemes.

External attacks	Pearson’s correlation coefficient (PCC) values						
	Run et al. (2012)	Run et al. (2012)	Mokashi et al. (2022)	Emir & Eskicioglu (2004) (DWT-SVD)	Alexander, Scott & Ahmet (2006) (DCT-SVD)	Justin et al. (2016) (DFT-SVD)	Proposed scheme
Gaussian noise with noise ratio 0.001	0.83	0.83	0.84	0.97	0.99	0.89	0.93
Gaussian Filtering 3 × 3	0.98	0.97	0.99	0.99	0.99	0.87	0.99
Gaussian filtering 5 × 5	0.98	0.97	0.99	0.99	0.99	0.87	0.99
Gaussian filtering 7 × 7	0.98	0.97	0.97	0.99	0.99	0.96	0.99
Histogram equalization	0.93	0.90	0.57	0.92	0.97	0.98	0.98
JPEG compression with 75% quality	0.44	0.21	0.92	0.85	0.98	0.97	0.98
JPEG Compression with 50% quality	0.44	0.22	0.93	0.85	0.95	0.92	0.95
Rotation 10°	0.64	0.53	0.93	0.93	0.91	0.96	0.96
Rotation 20°	0.59	0.51	0.89	0.91	0.93	0.94	0.94
Image resizing 512–>256	0.96	0.91	0.64	0.98	0.95	0.46	0.95
Image resizing 512–>1,024	0.98	0.91	0.95	0.98	0.97	0.97	0.97
Unsharp masking	0.96	0.88	0.91	0.91	0.98	0.74	0.98
Gamma correction $\gamma=0.8$	0.91	0.92	0.93	0.92	0.97	0.94	0.97
Gamma correction $\gamma=0.6$	0.91	0.97	0.61	0.95	0.96	0.98	0.98
Impulse noise 1%	0.67	0.65	0.91	0.88	0.95	0.63	0.94
Impulse noise 5%	0.36	0.35	0.82	0.58	0.64	0.36	0.84
Row flipping	0.62	0.82	0.83	0.95	0.94	0.80	0.95
Column flipping	0.64	0.85	0.74	0.96	0.95	0.81	0.95
Pixelate with 2 × 2 tiles	0.81	0.51	0.89	0.98	0.98	0.99	0.99
Pixelate with 4 × 4 tiles	0.53	0.54	0.85	0.57	0.94	0.43	0.94

**Table 7:** PCC analysis of extracted Circle watermark image from Man cover image impinged with different external attacks by various schemes.

Potential attacks	Pearson’s correlation coefficient (PCC) values						
	Run et al. (2012)	Run et al. (2012)	Mokashi et al. (2022)	Emir & Eskicioglu (2004) (DWT-SVD)	Alexander, Scott & Ahmet (2006) (DCT-SVD)	Justin et al. (2016) (DFT-SVD)	Proposed scheme
Gaussian noise with noise ratio 0.001	0.83	0.82	0.91	0.97	0.99	0.89	0.99
Gaussian filtering 3 × 3	0.94	0.95	0.99	0.97	0.99	0.74	0.99
Gaussian filtering 5 × 5	0.94	0.95	0.97	0.97	0.98	0.73	0.98
Gaussian filtering 7 × 7	0.94	0.95	0.97	0.97	0.98	0.73	0.98
Histogram equalization	0.96	0.89	0.98	0.98	0.91	0.99	0.99
JPEG compression with 75% quality	0.06	0.61	0.79	0.67	0.84	0.83	0.84
JPEG compression with 50% quality	0.04	0.61	0.74	0.67	0.83	0.82	0.83
Rotation 10^o^	0.61	0.56	0.97	0.95	0.96	0.99	0.98
Rotation 20^o^	0.57	0.52	0.94	0.93	0.95	0.96	0.96
Image resizing 512->256	0.51	0.85	0.65	0.94	0.89	0.93	0.93
Image resizing 512->1,024	0.95	0.91	0.91	0.99	0.98	0.98	0.98
Unsharp masking	0.91	0.81	0.94	0.91	0.96	0.96	0.96
Gamma correction $\gamma=0.8$	0.91	0.97	0.98	0.98	0.96	0.97	0.99
Gamma correction $\gamma=0.6$	0.95	0.89	0.85	0.98	0.99	0.91	0.99
Impulse noise 1%	0.64	0.65	0.92	0.87	0.95	0.59	0.95
Impulse noise 5%	0.33	0.41	0.86	0.58	0.67	0.33	0.72
Row flipping	0.67	0.93	0.85	0.96	0.96	0.97	0.97
Column flipping	0.65	0.95	0.95	0.97	0.91	0.98	0.98
Pixelate with 2 × 2 tiles	0.51	0.43	0.97	0.97	0.98	0.46	0.98
Pixelate with 4 × 4 tiles	0.2	0.42	0.91	0.38	0.89	0.40	0.95

**Table 8:** PCC analysis of extracted Cameraman watermark image from Barbara cover image impinged with different external attacks by various schemes.

Potential attacks	Pearson’s correlation coefficient (PCC) values						
	Run et al. (2012)	Run et al. (2012)	Mokashi et al. (2022)	Emir & Eskicioglu (2004) (DWT-SVD)	Alexander, Scott & Ahmet (2006) (DCT-SVD)	Justin et al. (2016) (DFT-SVD)	Proposed scheme
Gaussian noise with noise ratio 0.001	0.86	0.86	0.98	0.98	0.98	0.94	0.99
Gaussian filtering 3 × 3	0.97	0.94	0.96	0.97	0.98	0.71	0.99
Gaussian filtering 5 × 5	0.97	0.93	0.96	0.96	0.98	0.71	0.98
Gaussian filtering 7 × 7	0.91	0.93	0.96	0.95	0.96	0.71	0.96
Histogram equalization	0.97	0.82	0.84	0.96	0.99	0.97	0.99
JPEG compression with 75% quality	0.24	0.27	0.90	0.82	0.92	0.91	0.92
JPEG compression with 50% quality	0.23	0.27	0.87	0.82	0.90	0.89	0.90
Rotation 10°	0.63	0.59	0.89	0.98	0.95	0.98	0.98
Rotation 20°	0.59	0.55	0.95	0.95	0.91	0.96	0.96
Image resizing 512–>256	0.51	0.79	0.92	0.98	0.91	0.95	0.95
Image resizing 5,127>1,024	0.96	0.97	0.94	0.99	0.99	0.96	0.94
Unsharp masking	0.95	0.74	0.90	0.97	0.98	0.75	0.86
Gamma correction $\gamma=0.8$	0.99	0.99	0.96	1.00	1.00	0.99	0.99
Gamma correction $\gamma=0.6$	0.97	0.96	0.70	0.99	0.99	0.96	0.99
Impulse noise 1%	0.69	0.69	0.94	0.93	0.96	0.74	0.95
Impulse noise 5%	0.39	0.37	0.63	0.64	0.73	0.40	0.89
Row flipping	0.69	0.74	0.73	0.92	0.94	0.96	0.96
Column flipping	0.63	0.68	0.92	0.94	0.95	0.95	0.95
Pixelate with 2 × 2 tiles	0.55	0.91	0.96	0.97	0.94	0.76	0.97
Pixelate with 4 × 4 tiles	0.32	0.52	0.84	0.44	0.87	0.15	0.87

**Table 9:** BER and NCC analysis of extracted Rice watermark image from Lena cover image impinged with different external attacks by various schemes.

External attacks	Emir & Eskicioglu (2004) (DWT-SVD)		Alexander, Scott & Ahmet (2006) (DCT-SVD)		Justin et al. (2016) (DFT-SVD)		Proposed scheme	
	BER	NCC	BER	NCC	BER	NCC	BER	NCC
Gaussian noise with noise ratio 0.001	36.684	0.964	35.766	0.982	40.158	0.883	35.766	0.981
Gaussian filtering 3 × 3	39.597	0.992	38.897	0.992	44.014	0.867	38.797	0.994
Gaussian filtering 5 × 5	39.091	0.991	39.291	0.992	43.693	0.869	38.791	0.994
Gaussian filtering 7 × 7	39.591	0.99	39.591	0.984	43.793	0.873	38.791	0.994
Histogram equalization	42.075	0.976	39.675	0.970	40.784	0.969	39.464	0.958
JPEG compression with 75% quality	29.618	0.852	25.383	0.988	26.534	0.980	25.273	0.980
JPEG compression with 50% quality	35.501	0.861	31.067	0.989	31.637	0.974	30.063	0.989
Rotation 10°	41.634	0.905	39.802	0.878	41.434	0.932	39.232	0.926
Rotation 20°	34.099	0.958	33.819	0.958	32.599	0.961	32.479	0.961
Image resizing 512–>256	36.287	0.983	39.685	0.970	39.671	0.860	36.775	0.981
Image resizing 512–>1,024	32.102	0.989	33.095	0.972	32.702	0.985	32.375	0.969
Unsharp masking	38.485	0.923	38.485	0.989	47.314	0.749	38.454	0.976
Gamma correction $\gamma=0.8$	38.275	0.913	38.585	0.945	39.817	0.907	38.161	0.945
Gamma correction $\gamma=0.6$	39.524	0.819	38.485	0.825	38.165	0.844	39.410	0.813
Impulse noise 1%	36.429	0.919	33.877	0.991	44.586	0.659	35.378	0.937
Impulse noise 5%	50.268	0.682	38.275	0.790	59.361	0.425	37.775	0.989
Row flipping	30.837	0.977	30.947	0.969	29.974	0.982	30.637	0.980
Column flipping	33.979	0.995	33.989	0.976	32.032	0.983	33.323	0.976
Pixelate with 2 × 2 tiles	35.163	0.991	35.973	0.978	51.292	0.535	35.413	0.985
Pixelate with 4 × 4 tiles	54.542	0.597	39.394	0.993	60.950	0.456	38.994	0.984

**Table 10:** BER and NCC analysis of extracted Circle watermark image from Man cover image impinged with different external attacks by various schemes.

External attacks	Emir & Eskicioglu (2004) (DWT-SVD)		Alexander, Scott & Ahmet (2006) (DCT-SVD)		Justin et al. (2016) (DFT-SVD)		Proposed Scheme	
	BER	NCC	BER	NCC	BER	NCC	BER	NCC
Gaussian noise with noise ratio 0.001	36.684	0.964	35.766	0.982	40.158	0.883	37.721	0.931
Gaussian filtering 3 × 3	39.597	0.992	38.897	0.992	44.014	0.867	38.797	0.994
Gaussian filtering 5 × 5	39.091	0.991	39.291	0.992	43.693	0.869	38.791	0.994
Gaussian filtering 7 × 7	39.591	0.990	39.591	0.984	43.793	0.873	38.791	0.994
Histogram equalization	40.075	0.926	39.675	0.970	45.784	0.799	39.464	0.958
JPEG compression with 75% quality	29.618	0.852	25.383	0.988	26.534	0.980	25.773	0.980
JPEG compression with 50% quality	35.501	0.861	30.067	0.996	31.637	0.974	30.637	0.996
Rotation 10°	41.634	0.905	39.802	0.878	41.434	0.932	39.232	0.936
Rotation 20°	34.099	0.958	33.819	0.958	32.599	0.961	32.514	0.961
Image resizing 512->256	33.287	0.983	39.685	0.970	39.671	0.860	38.775	0.981
Image resizing 512–>1,024	32.202	0.999	33.095	0.972	32.702	0.985	32.375	0.999
Unsharp masking	38.485	0.963	38.485	0.989	47.314	0.749	38.454	0.976
Gamma correction $\gamma=0.8$	38.275	0.953	38.585	0.936	39.817	0.907	38.161	0.945
Gamma correction $\gamma=0.6$	39.524	0.819	38.485	0.825	38.165	0.844	39.410	0.813
Impulse noise 1%	36.429	0.919	33.877	0.991	44.586	0.659	35.378	0.937
Impulse noise 5%	50.268	0.682	38.275	0.790	59.361	0.425	37.775	0.989
Row flipping	30.837	0.977	30.947	0.969	29.974	0.982	30.637	0.980
Column flipping	33.979	0.995	33.989	0.976	32.032	0.983	33.323	0.976
Pixelate with 2 × 2 tiles	35.163	0.971	35.973	0.978	51.292	0.535	35.013	0.985
Pixelate with 4 × 4 tiles	54.542	0.597	39.394	0.993	60.950	0.456	38.994	0.995

**Table 11:** BER and NCC analysis of extracted Cameraman watermark image from Barbara cover image impinged with different external attacks by various schemes.

External attacks	Emir & Eskicioglu (2004) (DWT-SVD)		Alexander, Scott & Ahmet (2006) (DCT-SVD)		Justin et al. (2016) (DFT-SVD)		Proposed scheme	
	BER	NCC	BER	NCC	BER	NCC	BER	NCC
Gaussian Noise with noise ratio 0.001	36.684	0.964	35.766	0.982	40.158	0.883	37.721	0.931
Gaussian filtering 3 × 3	39.597	0.992	38.897	0.992	44.014	0.867	38.797	0.994
Gaussian filtering 5 × 5	39.091	0.991	39.291	0.992	43.693	0.869	38.791	0.994
Gaussian filtering 7 × 7	39.591	0.990	39.591	0.984	43.793	0.873	38.791	0.994
Histogram equalization	39.075	0.976	39.675	0.970	38.784	0.979	38.764	0.978
JPEG compression with 75% quality	29.618	0.852	25.383	0.988	26.534	0.980	25.773	0.983
JPEG compression with 50% quality	35.501	0.861	30.067	0.996	31.637	0.974	30.637	0.989
Rotation 10°	41.634	0.905	42.802	0.878	41.434	0.932	39.232	0.926
Rotation 20°	34.099	0.958	43.819	0.958	32.599	0.961	32.979	0.958
Image resizing 512–>256	32.287	0.983	39.685	0.970	39.671	0.860	38.775	0.981
Image resizing 512–>1,024	32.302	0.984	33.095	0.972	32.702	0.985	32.375	0.969
Unsharp masking	38.485	0.993	38.485	0.999	47.314	0.749	38.454	0.999
Gamma correction $\gamma=0.8$	38.275	0.953	38.585	0.936	39.817	0.907	38.161	0.945
Gamma correction $\gamma=0.6$	39.524	0.819	38.485	0.825	38.765	0.844	38.41	0.853
Impulse noise 1%	36.429	0.919	33.877	0.991	44.586	0.659	33.878	0.991
Impulse noise 5%	50.268	0.682	38.275	0.790	59.361	0.425	37.775	0.989
Row flipping	30.837	0.977	30.947	0.969	29.974	0.982	30.637	0.980
Column flipping	33.979	0.995	33.989	0.976	32.032	0.995	33.323	0.976
Pixelate with 2 × 2 tiles	35.163	0.991	35.973	0.978	51.292	0.535	35.413	0.985
Pixelate with 4 × 4 tiles	54.542	0.597	39.394	0.993	60.950	0.456	38.994	0.993

From Table 9, it is clear that Emir & Eskicioglu (2004), Alexander, Scott & Ahmet (2006), Justin et al. (2016) algorithms produce best results for three, two and two potential attacks respectively in terms of BER while the proposed method produces best results for 13 image processing attacks. Table 10 demarcates that Emir & Eskicioglu (2004), Alexander, Scott & Ahmet (2006), Justin et al. (2016) algorithms produce best results for two, four and three potential attacks respectively in terms of BER while the proposed method produces best results for 11 image processing attacks. Table 11 shows that Emir & Eskicioglu (2004), Alexander, Scott & Ahmet (2006), Justin et al. (2016) algorithms produce best BER results for three, four and three potential attacks respectively while the proposed method produces best results for 10 image processing attacks.

From  Table 9, it is clear that Emir & Eskicioglu (2004), Alexander, Scott & Ahmet (2006), Justin et al. (2016) algorithms produce best results for five, six and four potential attacks respectively in terms of NCC while the proposed method produces best results for seven image processing attacks. Table 10 shows that Emir & Eskicioglu (2004), Alexander, Scott & Ahmet (2006), Justin et al. (2016) algorithms produce best results for three, five and four potential attacks respectively in terms of NCC while the proposed method produces best results for 12 image processing attacks. Table 11 shows that Emir & Eskicioglu (2004), Alexander, Scott & Ahmet (2006), Justin et al. (2016) algorithms produce best NCC results for five, six and four potential attacks respectively while the proposed method produces best results for nine image processing attacks. It is clear from Tables 6–11 that the results of proposed method is consistent in providing better robustness against all potential attacks although it fails in some cases to produce best results in terms of PCC, BER and NCC values.

**Figure 6:**
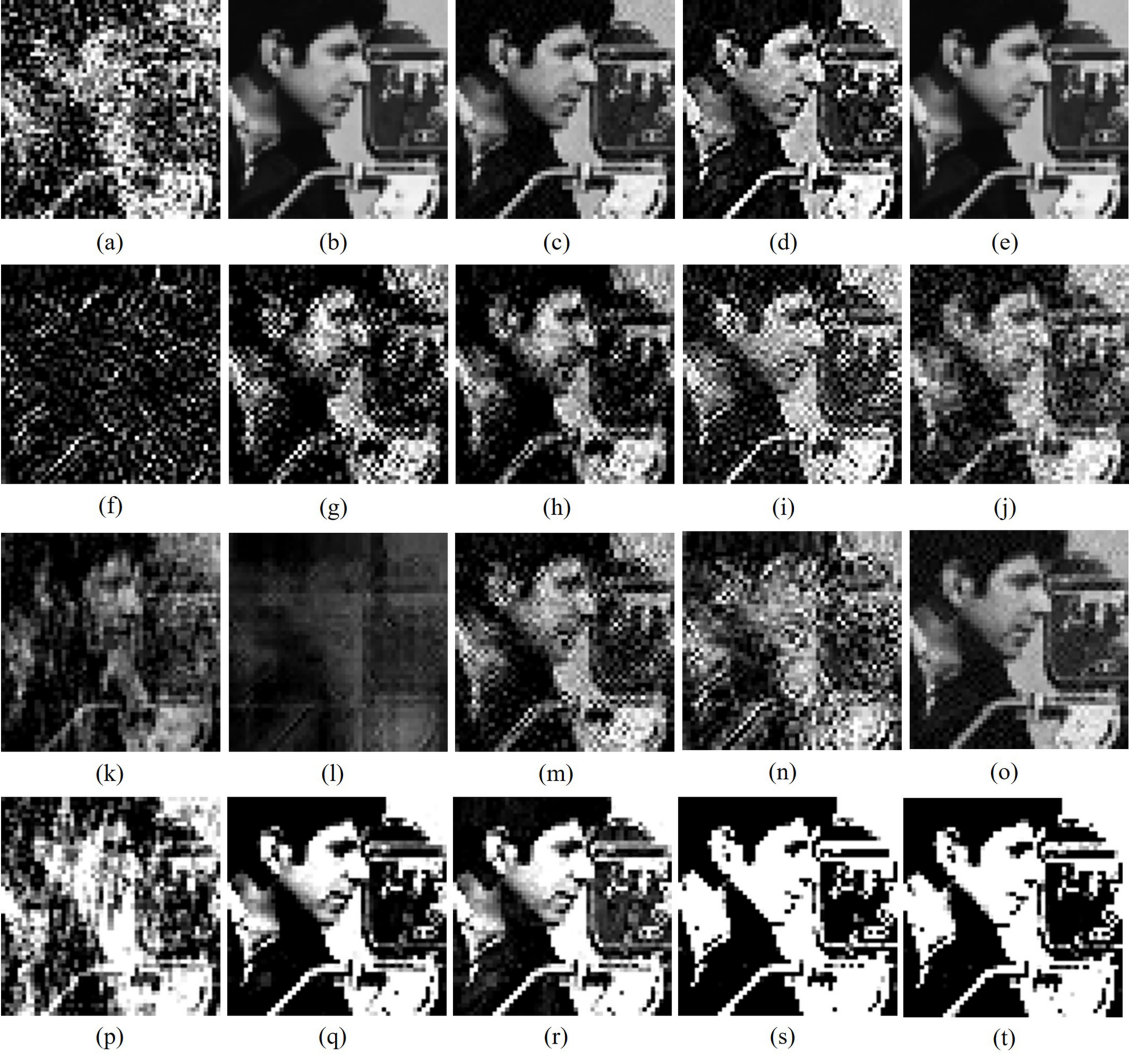
Cropped versions of Cameraman watermark extracted by different algorithms from watermarked Barbara image impinged with different attacks. Different algorithms: column 1 (A, F, K, P): Run et al. (2012), column 2 (B, G, L, Q): Emir & Eskicioglu (2004), column 3 (C, H, M, R): Alexander, Scott & Ahmet (2006), column 4 (D, I, N, S): Justin et al. (2016) and column 5 (E, J, O, T): proposed schemes. Different attacks: row 1 (A–E): Gaussian noise with noise ratio 0.001, row 2 (F–J): rotation 45O, row 3 (K–O): Gaussian filter 7 × 7, row 4 (P–T): histogram equalization. Test images are from https://ccia.ugr.es/cvg/CG/base.htm open database.

**Figure 7:**
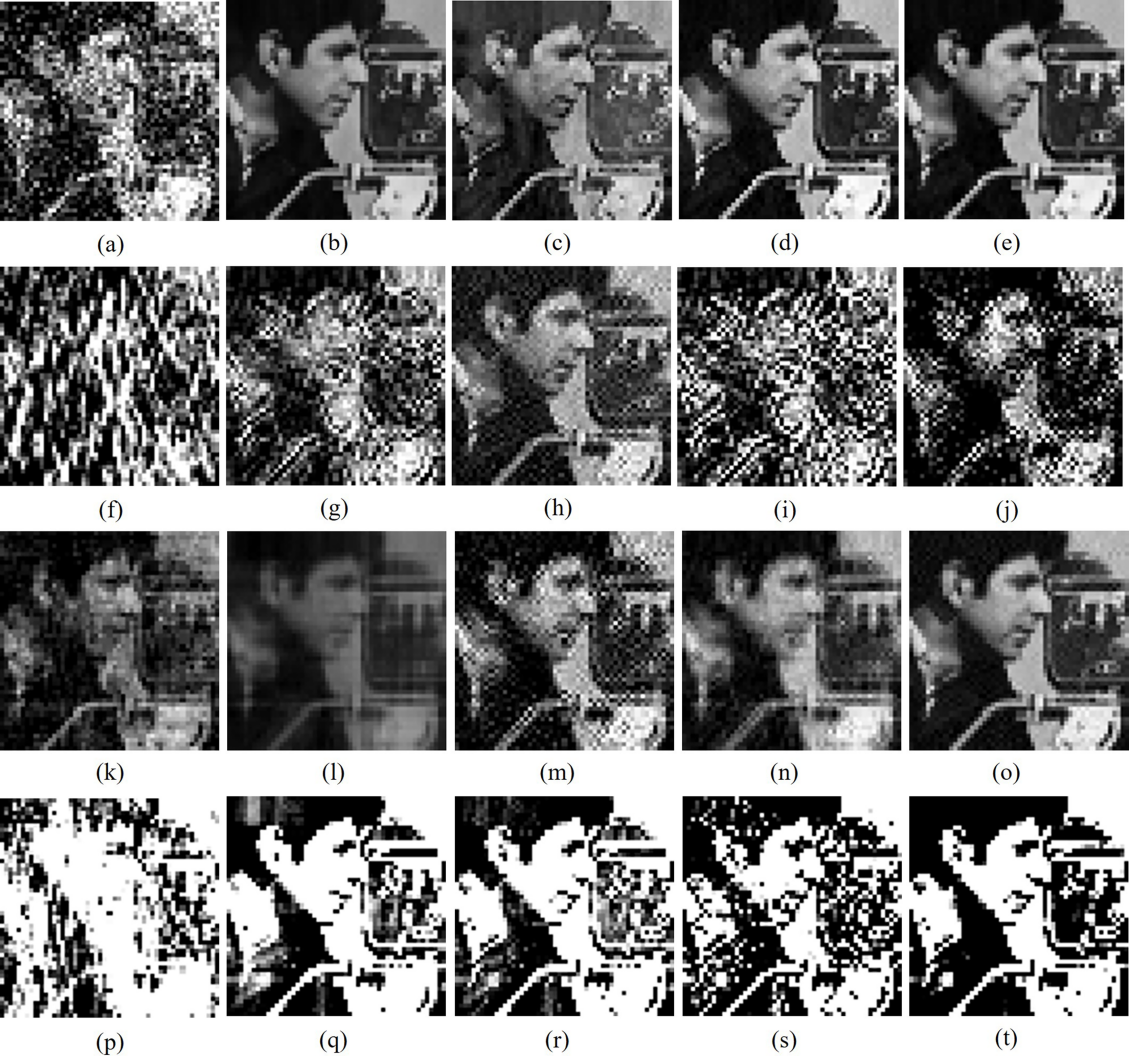
Cropped versions of Cameraman watermark extracted by different algorithms from watermarked Barbara image impinged with different attacks. Different algorithms: column 1 (A, F, K, P): Run et al. (2012), column 2 (B, G, L, Q): Emir & Eskicioglu (2004), column 3 (C, H, M, R): Alexander, Scott & Ahmet (2006), column 4 (D, I, N, S): Justin et al. (2016) and column 5 (E, J, O, T): proposed schemes. Different attacks: row 1 (A–E): JPEG 50% quality, row 2 (F–J): image resizing (512-256-512), row 3 (K–O): image resizing (512-1024-512), row 4 (P–T): unsharp masking. Test images are from https://ccia.ugr.es/cvg/CG/base.htm open database.

**Figure 8:**
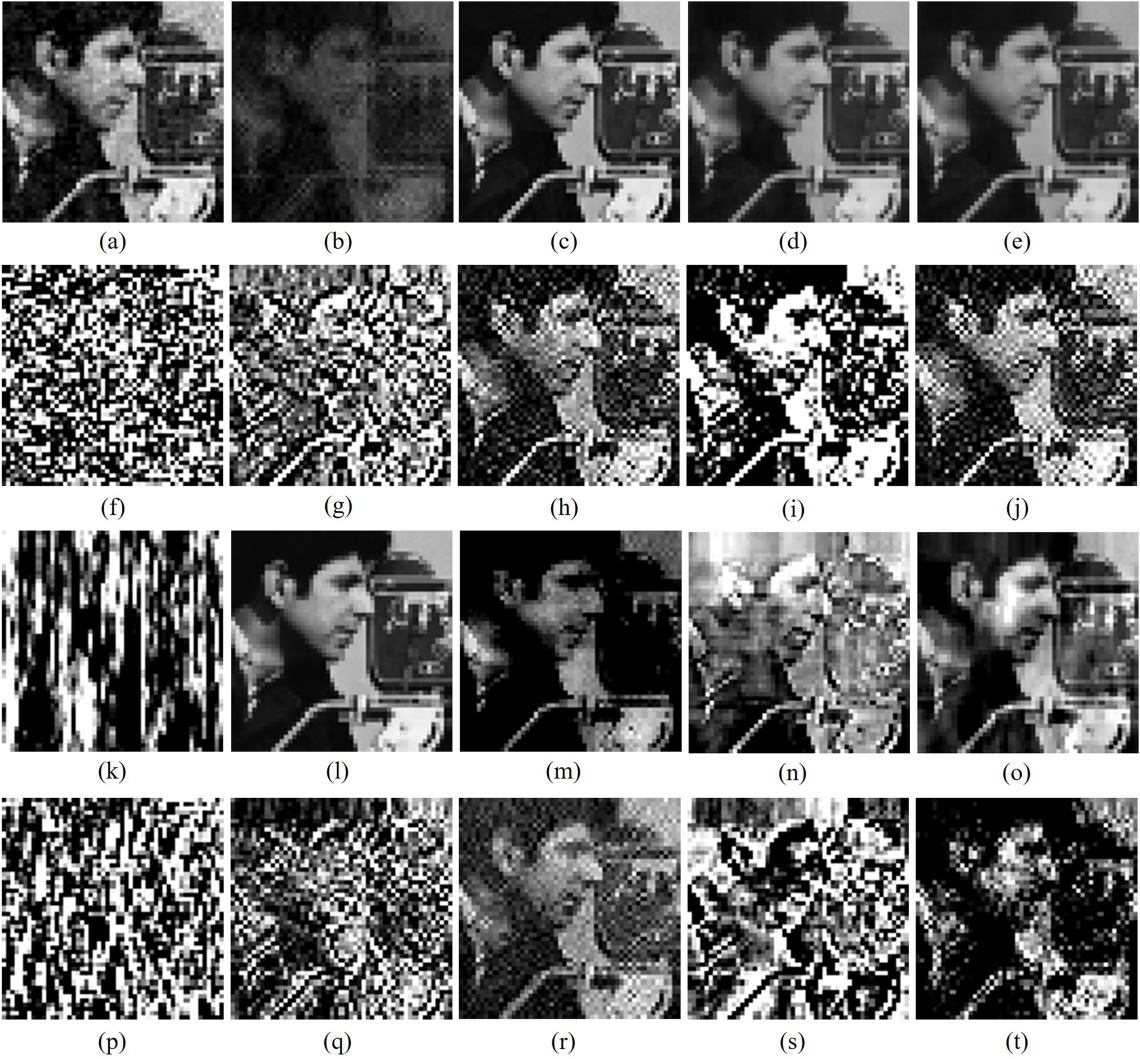
Cropped versions of Cameraman watermark extracted by different algorithms from watermarked Barbara image impinged with different attacks. Different algorithms: column 1 (A, F, K, P): Run et al. (2012), column 2 (B, G, L, Q): Emir & Eskicioglu (2004), column 3 (C, H, M, R): Alexander, Scott & Ahmet (2006), column 4 (D, I, N, S): Justin et al. (2016) and column 5: proposed schemes. Different attacks: row 1 (A-E): contrast stretching, row 2 (F-J): impulse noise with 5% noise, row 3 (K-O): column flip, row 4 (P-T): pixelate 4 × 4. Test images are from https://ccia.ugr.es/cvg/CG/base.htm open database.

Figures 6–8 makes visual analysis of watermark images reproduced by various algorithms from cover images interrupted with different external attacks. From Tables 6–11 and from Figs. 6–8, it can be clearly noted that unlike other algorithms, the new watermarking scheme makes consistent performance for majority of attacks and provides better quantitative values than other algorithms for majority of external attacks by utilizing advantages of DFT, DCT and SVD transforms while attaching secret contents in cover images.

## Conclusions

The article presented a new non-blind watermarking algorithm that utilized the combined merits of DFT, DCT and SVD transforms. With the embedding of watermark replicas to all cover image frequencies, the new scheme provides improved resistance towards external potential attacks since the algorithm has more possibility for protecting some watermark replicas although the potential attacks may damage other replicas attached in other carrier image frequencies. As the algorithm utilizes the collective advantages of DFT, DCT and SVD transforms while attaching secret contents in cover images, it shows better robustness and imperceptibility capabilities than other algorithms used in the study. The experimental results based on subjective and objective metrics on various test images with different test conditions showed that the proposed non-blind algorithm exhibits better consistency by producing high visual quality images offering better resistance against external attacks.

### Future scope

The new scheme is designed for performing consistently to provide acceptable watermark though the cover image is attacked with different external attacks. Future algorithms can look up on extending the new scheme to provide better quality watermarks for majority of external attacks. Intelligent hybrid solutions can be looked up on with better homogeneity analysis of the carrier and watermark images to achieve better quality watermarked image with comparatively less visual distortions.

## Supplemental Information

10.7717/peerj-cs.1427/supp-1 Supplemental Information 1 Computer Code (Matlab) with Test Images. The main program, and the subfunctions and programs (bloc.m, extract.m, farng.m, iarng.m, izigzag.m, rebloc1.m, watermkg.m, zigzag.m) required for calculations. The carrier and watermark image files are: barb.gif, cameraman.tif.
The images used in this study are non-copyrighted standard image processing test images widely available through many open image databases like http://sipi.usc.edu/database/ and http://www.imageprocessingplace.com/root_files_V3/image_databases.htm.

## Additional Information and Declarations

### Data Availability

The following information was supplied regarding data availability: The MatLab code is available in the Supplemental File.
