# Supplementary figures and images for: An effective digital image watermarking scheme incorporating DCT, DFT and SVD transformations
Source: PeerJ Comput Sci. 2023 Jul 10;9:e1427. doi: 10.7717/peerj-cs.1427 (PMC10403172; doi:10.7717/peerj-cs.1427)

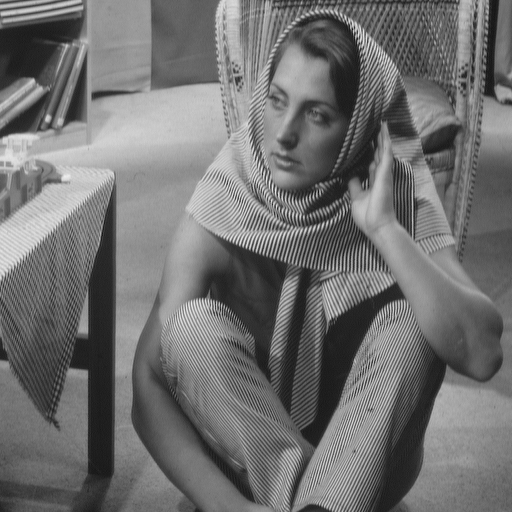

Supplement: Supplemental Information 1 — The main program, and the subfunctions and programs (bloc.m, extract.m, farng.m, iarng.m, izigzag.m, rebloc1.m, watermkg.m, zigzag.m) required for calculations. The carrier and watermark image files are: barb.gif, cameraman.tif. The images used in this study are non-copyrighted standard image processing test images widely available through many open image databases like http://sipi.usc.edu/database/ and http://www.imageprocessingplace.com/root_files_V3/image_databases.htm. [file peerj-cs-09-1427-s001.zip › Code/barb.gif]

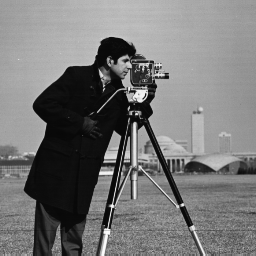

Supplement: Supplemental Information 1 — The main program, and the subfunctions and programs (bloc.m, extract.m, farng.m, iarng.m, izigzag.m, rebloc1.m, watermkg.m, zigzag.m) required for calculations. The carrier and watermark image files are: barb.gif, cameraman.tif. The images used in this study are non-copyrighted standard image processing test images widely available through many open image databases like http://sipi.usc.edu/database/ and http://www.imageprocessingplace.com/root_files_V3/image_databases.htm. [file peerj-cs-09-1427-s001.zip › Code/cameraman.tif]
